# Association between Pre-Diagnostic Serum Bile Acids and Hepatocellular Carcinoma: The Singapore Chinese Health Study

**DOI:** 10.3390/cancers13112648

**Published:** 2021-05-28

**Authors:** Claire E. Thomas, Hung N. Luu, Renwei Wang, Guoxiang Xie, Jennifer Adams-Haduch, Aizhen Jin, Woon-Puay Koh, Wei Jia, Jaideep Behari, Jian-Min Yuan

**Affiliations:** 1 Department of Epidemiology, Graduate School of Public Health, University of Pittsburgh, Pittsburgh, PA 15261, USA; cet53@pitt.edu (C.E.T.); HNL11@pitt.edu (H.N.L.); 2 UPMC Hillman Cancer Center, University of Pittsburgh, Pittsburgh, PA 15232, USA; wangr2@upmc.edu (R.W.); adamshaduchj@upmc.edu (J.A.-H.); jab31@pitt.edu (J.B.); 3 University of Hawaii Cancer Center, Honolulu, HI 96813, USA; xie26guo@163.com (G.X.); weijia1@hkbu.edu.hk (W.J.); 4 Healthy Longevity Translational Research Programme, Yong Loo Lin School of Medicine, National University of Singapore, Singapore 117545, Singapore; jinaz@nus.edu.sg (A.J.); kohwp@nus.edu.sg (W.-P.K.); 5 Department of Medicine, Division of Gastroenterology, Hepatology, and Nutrition, School of Medicine, University of Pittsburgh, Pittsburgh, PA 15261, USA

**Keywords:** serum bile acids, hepatocellular carcinoma, molecular epidemiology, metabolism, liver

## Abstract

**Simple Summary:**

Hepatocellular carcinoma (HCC) is a common cancer with poor prognosis. The increasing incidence rate of HCC in developed countries has been linked to increasing prevalence of metabolic dysfunction-associated fatty liver disease, which has characteristics of altered bile acid metabolism that may predate hepatocarcinogenesis. The aim of the present study was to assess the association of circulating bile acid levels in pre-diagnostic serum with the risk of developing HCC in a general population in Singapore. Primary conjugated bile acids were most strongly associated with increased risk of HCC whereas the ratios of secondary over primary bile acids were significantly associated with reduced risk. These results support a contributing role of dysmetabolism of bile acids in the development of HCC. The modulation of bile acid metabolism through alteration of gut microbiota may be an effective strategy for primary prevention against HCC in individuals with metabolic dysfunction-associated fatty liver disease.

**Abstract:**

Hepatocellular carcinoma (HCC) is a commonly diagnosed malignancy with poor prognosis. Rising incidence of HCC may be due to rising prevalence of metabolic dysfunction-associated fatty liver disease, where altered bile acid metabolism may be implicated in HCC development. Thirty-five bile acids were quantified using ultra-performance liquid chromatography triple-quadrupole mass spectrometry assays in pre-diagnostic serum of 100 HCC cases and 100 matched controls from the Singapore Chinese Health Study. Conditional logistic regression was used to assess associations for bile acid levels with risk of HCC. Conjugated primary bile acids were significantly elevated whereas the ratios of secondary bile acids over primary bile acids were significantly lower in HCC cases than controls. The respective odds ratios and 95% confidence intervals of HCC were 6.09 (1.75–21.21) for highest vs. lowest tertile of cholic acid species and 30.11 (5.88–154.31) for chenodeoxycholic acid species. Doubling ratio of taurine-over glycine-conjugated chenodeoxycholic acid was associated significantly with 40% increased risk of HCC whereas doubling ratio of secondary over primary bile acid species was associated with 30–40% reduced risk of HCC. In conclusion, elevated primary bile acids and taurine over glycine-conjugated ratios were strongly associated with HCC risk whereas the ratios of secondary bile acids over primary bile acids were inversely associated with HCC risk.

## 1. Introduction 

Liver cancer is the sixth most commonly diagnosed cancer and fourth most common cause of cancer-related death globally [1]. Among subtypes of primary liver cancer, hepatocellular carcinoma (HCC) is most common, accounting for 80–90% of primary liver cancer cases across different populations [2,3]. The major risk factors for HCC are chronic infection with hepatitis B virus (HBV) and/or hepatitis C virus (HCV), alcohol abuse, and dietary exposure to aflatoxin B_1_ [4,5,6]. HBV vaccination has resulted in significantly decreased prevalence of chronic hepatitis B in younger generations worldwide whereas available curative therapies for chronic infection with HCV would diminish the role of HCV in the development of HCC. Given the diminishing contributing role of HBV and HCV to HCC development, non-alcoholic fatty liver disease (NAFLD), recently redefined under the term metabolic dysfunction-associated fatty liver disease (MAFLD) [7], has emerged as an important risk factor for HCC. The increasing incidence and mortality of HCC in the US [8] and globally [6,9] could be due to the rising prevalence of MAFLD, which is highly associated with obesity, diabetes and metabolic syndrome [10,11]. MAFLD encompasses a spectrum of disease severity ranging from simple steatosis to non-alcoholic steatohepatitis (NASH), which can progress to fibrosis, cirrhosis, and HCC. What factors determine and enhance the progression of MAFLD to HCC remain to be clarified. Emerging data suggest that altered gut microbiome (i.e., dysbiosis) due to dietary and other lifestyle exposures may play a significant role in the development of various liver diseases including MAFLD and HCC [12,13]. One of the direct links from the gut microbiome to the host liver is through microbial-produced secondary bile acids via enterohepatic circulation [14].

Primary bile acids, i.e., cholic acid (CA) and chenodeoxycholic acid (CDCA), are synthesized from cholesterol in the liver [14,15,16]. In humans, the classical pathway, regulated by cytochrome P450 7-alpha-hydroxylase (CYP7A1) [15], is responsible for the synthesis of both CA and CDCA whereas the alternative pathway via CYP7B1 and CYP27A1 produces only CDCA [15]. Most CA and CDCA are conjugated with either glycine to form glyco-CA (GCA) and glyco-CDCA (GCDCA) or taurine to form tauro-CA (TCA) and tauro-CDCA (TCDCA), respectively, in the liver before they are secreted in bile and stored in the gallbladder. After ingestion of food, bile is released into the small intestine where bile salts are deconjugated and facilitate the absorption and metabolism of lipids and fat-soluble vitamins. Approximately 95% of total primary bile acids are reabsorbed from the distal small intestine and recycled back to the liver through enterohepatic circulation [17]. The remaining primary bile acids (~5%) flow into the colon where gut microbiota alter the structures of primary bile acids to form secondary bile acids, CA to deoxycholic acid (DCA), and CDCA to lithocholic acid (LCA) and ursodeoxycholic acid (UDCA), respectively [14]. The majority of secondary bile acids are absorbed by colonocytes and transported via the portal vein to the liver where they are further metabolized, conjugated, and enter the enterohepatic circulation in the same way as the primary bile acids [15,16]. The human bile acid pool consists of a large proportion of primary bile acids and their conjugates with a relatively small proportion of secondary bile acids [15].

Increasing evidence in both humans and mice suggests an association between altered bile acids profile and HCC [18,19,20,21,22] and some studies have examined this association prospectively [23,24,25,26,27]. However, some of these previous studies have examined this association using samples collected after diagnosis of HCC [21,28,29,30], measured a limited number of bile acids, and/or were in selected study populations, such as among those with chronic hepatitis. Thus, the findings from these previous studies might be confounded by the disease status on the measurements of bile acids, have a narrow view of bile acids profile, or have limited generalizability to general populations. To overcome these potential limitations, we conducted a nested case–control study of HCC within a prospective cohort study consisting of individuals drawn from a general population, the Singapore Chinese Health Study, to comprehensively evaluate the associations for the metabolic profile of bile acids with the risk of developing HCC.

## 2. Methods

### 2.1. Study Population

The present study was established within the Singapore Chinese Health Study, which has been approved by the Institutional Review Boards of the National University of Singapore and the University of Pittsburgh. The present study was approved by the Institutional Review Board of the University of Pittsburgh.

The Singapore Chinese Health Study is a prospective population-based cohort study that recruited 63,257 Chinese men and women, aged 45–74 years, in Singapore, between April 1993 and December 1998 [31]. The eligible subjects had to be permanent residents of Singapore government-built housing and belong to one of two major Chinese dialect groups—Hokkien or Cantonese. At initial enrollment, an in-person interview was administered by a trained interviewer using a structured questionnaire for participant information on demographics and lifestyle characteristics. In addition, a validated semi-quantitative food frequency questionnaire was used to collect participant information on habitual dietary intake, including consumption of alcoholic beverages during the past 12 months [32]. Urine and blood samples were collected from 3% of randomly selected participants between April 1994 and December 1999. From July 1999 to December 2003, all surviving participants were contacted to update their lifestyle characteristics information by telephone and asked if they were willing to donate biospecimens. Urine and blood, or buccal sample if blood donation was declined, were collected from all consenting participants from January 2000 to April 2005. A total of 32,535 participants (approximately 60% of surviving participants) donated blood, buccal and/or urine samples for research. All components (buffy coat, plasma, red blood cells, and serum) of blood were separated within 4 h and multiple aliquots of blood components and urine samples were stored at −80 °C until analysis.

### 2.2. Ascertainment of Incident Cancer Cases and Death

All study participants were followed up annually for the incidence of cancer and death. Incident cancer cases were identified through linkage analysis with the nationwide Singapore Cancer Registry and deaths were ascertained via the Singapore Birth and Death Registry. The Singapore Cancer Registry has collected comprehensive information on cancer diagnoses since 1968 [33]. The follow-up for cancer incidence and death was virtually complete. To date, 56 participants (<0.1%) have been cumulatively lost to follow-up.

### 2.3. Nested Case–Control Study of Hepatocellular Carcinoma

As of 31 December 2015, we identified 216 incident HCC cases among participants who provided a pre-diagnostic serum sample. For the present study, we chose the first 100 incident HCC cases. We randomly selected one control subject per case among all potentially eligible subjects with available baseline serum samples. The control had to be alive and free of cancer at the time of cancer diagnosis for the index case and was individually matched to the index case by age at enrollment (±3 years), gender, dialect group (Hokkien, Cantonese), date of biospecimen collection (±6 months), and date of baseline interview (±2 year).

### 2.4. Measurement of Serum Bile Acids and Hepatitis B Virus

Serum samples of all selected study participants were assayed for bile acids (BAs) using ultra-performance liquid chromatography triple-quadrupole mass spectrometry (UPLC-TQMS) as described previously [34,35,36] at the Jia Lab at the University of Hawai’i Cancer Center. Briefly, all bile acid standards were acquired from TRC Chemicals (Toronto, ON, Canada) and Steraloids, Inc. (Newport, RI, USA). Nine stable isotope-labeled standards including CA-d4, GCA-d4, DCA-d4, GDCA-d4, TCA-d4, LCA-d4, UDCA-d4, GCDCA-d4, and TCDCA-d9 used as internal standard (IS) were obtained from C/D/N Isotopes, Inc. (Pointe-Claire, QC, Canada) and Steraloids, Inc. (Newport, RI, USA). The standards and IS were accurately weighed and prepared in methanol at a concentration of 5.0 mM (stock solution). Further dilution was performed to obtain a series of calibration concentrations of 2000, 400, 160, 32, 12.8, 2.5, and 1 nM with methanol/water (50/50, *v*/*v*). IS concentrations were kept constant at all the calibration points at 100 nM. Each 100 µL of serum or standard solution in BA-free matrix was lyophilized to dry powder using a freeze dryer, and the residue reconstituted in 1:1 (*v*/*v*) mobile phase B (acetonitrile/methanol = 95:5, *v*/*v*) and mobile phase A (water with formic acid) and centrifuged at 13,500× *g* and 4 °C for 20 min. The supernatant was transferred to a 96-well plate and was analyzed with a UPLC-TQMS system (ACQUITY UPLC-Xevo TQ-S, Waters Corp., Milford, MA, USA). All chromatographic separations were performed with an Acquity UPLC C18 column (1.7 µm, 100 mm × 2.1 mm I.D.; Waters). The raw data was processed using the TargetLynx application manager (Waters Corp., Milford, MA, USA) to obtain calibration equations and the measured concentration of each BA in individual samples. The intra- and inter-batch CVs were less than 10% and the recovery rate was 95–110% for all BAs, as reported in a previous study [34]. The case/control statuses of the test samples were unknown to the laboratory personnel who performed the assays for bile acids.

Serological status of HBsAg and antibodies to HCV (anti-HCV) on all subjects included in the present studies were tested previously [37,38]. Briefly, the presence of HBsAg was determined by using a standard radioimmunoassay (AUSRIA, Abbott Laboratories, North Chicago, IL, USA), and anti-HCV using the ELISA version 2.0 kit (Ortho Diagnostic Systems, Raritan, NJ, USA), with confirmation of positive samples using the RIBA version 2.0 (Chiron, Emeryville, CA, USA).

### 2.5. Statistical Analysis

The names of all individual bile acids tested are listed in Supplementary Table S1 according to the nomenclature proposed by Hofmann et al. [39] Individual bile acids were grouped into different species based on the parent bile acid, origin (primary and secondary), and conjugation with taurine, glycine, and other compounds (e.g., glucuronide, sulfate). In addition, we created variables for the ratios of taurine-conjugated over glycine-conjugated bile acids and the ratios of secondary over primary bile acids at individual and species group levels, respectively, for statistical analysis.

The distributions of absolute concentrations and ratios of bile acids in serum were rightward skewed. Logarithmically transformed values were used in formal statistical testing, and geometric means and 95% confidence intervals (CIs) are presented. Differences in distributions of baseline characteristics between HCC cases and controls were determined by *t*-tests for continuous variables and Chi-square test for frequencies. The analysis of variance (ANOVA) method was used to examine the differences in absolute abundance of bile acids among controls by different levels of exposures such as alcohol intake, body mass index (BMI), diabetes, and HBsAg seropositivity status. Spearman correlation coefficients were calculated to assess the correlation between two individual bile acids in control subjects.

The conditional logistic regression method was used to calculate odds ratios (ORs) and 95% CIs for HCC associated with tertile and the doubling (log_2_) concentrations of bile acids or their ratios with the adjustment for covariates measured at time of blood collection, including HBsAg seropositivity status (positive or negative), BMI (kg/m^2^), smoking status (never, former, current), alcohol intake (0, <1, 1+ drinks per day), diabetes status (yes, no), and hours from last meal to blood draw (<3 h, 3– <6 h, 6+ hours). Tertiles were calculated based on the distribution of each variable among control subjects. Linear trend for HCC risk with levels of bile acids was tested based on the ordinal values of their tertiles. Sensitivity analyses were conducted among two subgroups: (1) the case–control pairs in which both the index case and the matched control did not test positive for both HBsAg and anti-HCV; and (2) the case–control pairs whose case was diagnosed at least two years after the baseline blood draw.

Statistical analyses were performed using SAS version 9.4 (SAS Institute Inc., Cary, NC, USA) and R version 3.6. All *p* values reported are two-sided, and *p* values less than 0.05 were considered statistically significant.

## 3. Results

The mean (standard deviation) age of HCC patients at diagnosis was 70.2 (7.6) years. Ages at baseline blood collection for both cases and controls were well matched (Table 1). In HCC cases, the mean (standard deviation) time interval from blood collection to HCC diagnosis was 4.3 (2.3) years. Compared to controls, HCC cases were more likely to be HBsAg positive and more likely to have a history of diabetes (Table 1). Cases and controls had comparable BMI, time interval from the last meal to blood draw, alcohol intake, and smoking status.

### 3.1. Serum Concentrations of Primary Bile Acids and HCC Risk

**Figure 1:**
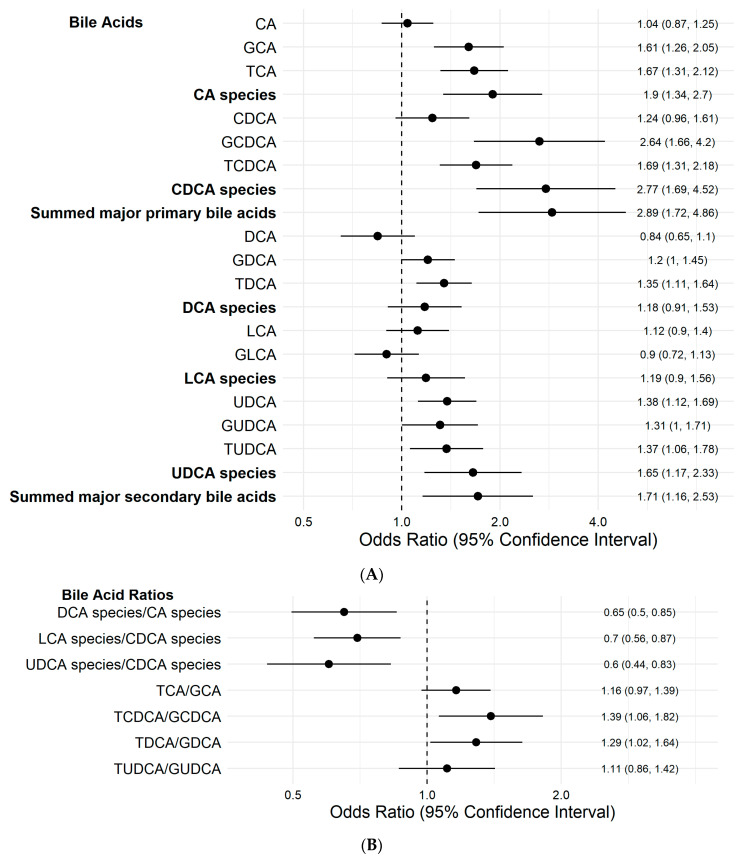
Odds ratios (95% confidence intervals) ^a^ of hepatocellular carcinoma associated with (**A**) doubling concentrations of bile acids, (**B**) doubling the ratio of secondary bile acid species over primary bile acids species and doubling the ratios of taurine-over glycine-conjugated bile acids, The Singapore Chinese Health Study. ^a^ Derived from conditional logistic regression models including the following covariates: HBsAg status, alcohol intake, cigarette smoking status, history of diabetes, body mass index, and time interval from the last meal to blood draw. Summed major primary bile acids: sum of CA species and CDCA species. Summed major secondary bile acids: sum of DCA species, LCA species, and UDCA species.

HCC cases had significantly higher concentrations of CA species and CDCA species and their sum than controls (Table 2). The ORs (95% CIs) for HCC for the highest relative to the lowest tertile of CA and CDCA species were 6.09 (1.75–21.21) and 30.11 (5.88–154.31), respectively (both *P*_trend_ < 0.001), after adjustment for HBsAg seropositivity status, alcohol intake, smoking status, diabetes status, BMI, and hours from the last meal to blood draw (Table 3). Doubling concentrations of CA and CDCA species were associated with a statistically significantly 90–177% increased risk of HCC (Figure 1A).

### 3.2. Serum Concentrations of Secondary Bile Acids and HCC Risk

HCC cases had higher levels of total secondary bile acids than controls (Table 2). HCC cases had significantly higher levels of the summed total secondary bile acid and UDCA species than controls. For relative abundance of the secondary bile acids, the ratios of the secondary bile acids over their parent primary bile acids were constructed. Specifically, the ratios of DCA over CA, LCA over CDCA, and UDCA over CDCA species were significantly lower in HCC cases than in controls (Table 2). Compared with the lowest tertile, ORs (95% CIs) of HCC for the highest tertile of the DCA/CA ratio, LCA/CDCA ratio, and UDCA/CDCA ratio were 0.37 (0.14–1.00), 0.27 (0.09–0.81), and 0.29 (0.10–0.82), respectively (Table 3). The risk of HCC was significantly decreased by 30–40% with doubling ratios of these secondary bile acids over their parent primary bile acids (Figure 1B).

### 3.3. Taurine-Conjugated and Glycine-Conjugated Bile Acids and HCC Risk

Both glycine- and taurine-conjugated major primary and secondary bile acids were significantly more elevated in HCC cases than in controls (Table 4). Compared with the lowest tertile, the highest tertile of glycine- and taurine-conjugated CA, CDCA, and DCA species were associated with 2.5- to 57.2-fold increased risk of HCC (Table 5).

The ratios of taurine-conjugated over glycine-conjugated CDCA (i.e., TCDCA/GCDCA ratio) and DCA (i.e., DCA/GDCA ratio) were significantly higher in HCC cases than in controls (Table 4). Compared with the lowest tertile, the highest tertile of these taurine-conjugated over glycine-conjugated bile acids was associated with 3 to 4 times increased risk of HCC (Table 5). Doubling the ratios of these taurine-over glycine-conjugated bile acids was significantly associated with 30–40% increased risk of HCC (Figure 1B).

### 3.4. Serum Concentrations of Other Minor Bile Acids and HCC Risk

Besides the major primary and secondary bile acids, we quantified additional minor bile acids (n = 21) in the serum of study subjects. Among them, serum concentrations of CDCA-24-glucuronide (CDCA-24G) were significantly more elevated in HCC cases than in controls (411.2 nM vs. 106.7 nM, *p* < 0.001) (Supplementary Table S2). Doubling concentrations of CDCA-24G was significantly associated with an OR of 1.55 (95% CI: 1.23–1.95) (Supplementary Table S3). Additionally, doubling concentrations of glycol-hyodeoxycholic acid (GHDCA) was significantly associated with an OR of 1.39 (95% CI: 1.00–1.92). No consistent statistically significant association was observed for other individual minor bile acids with the risk of HCC.

### 3.5. Correlation of Serum Bile Acids with Each Other and Other Covariates

Among all control subjects, the correlation was relatively high between GCA and GCDCA (Spearman *r* = 0.6, *p* < 0.001), but was modest between free CA and free CDCA (Spearman *r* = 0.3, *p*= 0.003), and TCA and TCDCA (Spearman *r* = 0.2, *p*= 0.049) (Supplementary Figure S1). TCDCA and GCDCA were the most strongly correlated bile acids (Spearman *r* = 0.8, *p* < 0.001). The secondary bile acids correlations with their corresponding parent primary bile acids varied; the correlation coefficient was 0.2 between DCA and CA (*p* = 0.079) and 0.5 between UDCA and CDCA (*p* < 0.001), but null between LCA and CDCA (Spearman *r* = 0).

We did not find any impact of age, sex, alcohol intake, smoking status, and HBsAg seropositivity on serum concentrations of primary or secondary bile acid species among controls (Supplementary Table S4). The time interval between the last meal and blood draw was inversely correlated with levels of total primary bile acids, specifically CDCA species. Increasing BMI was correlated with increased levels of CA species, CDCA species, summed major primary species, and DCA species. Individuals with a history of diabetes had higher UDCA species than nondiabetics.

### 3.6. Sensitivity Analysis for Bile Acids and HCC Risk

To avoid the impact of underlying chronic liver disease progression on the synthesis and metabolism of bile acids, we repeated our analysis for the associations between various measurements of serum bile acids and the risk of HCC after excluding all case–control pairs where at least one subject tested positive for HBsAg or anti-HCV. Among the 53 case–control pairs who were not positive for HBsAg and anti-HCV, the bile acid–HCC risk associations remained similar to those observed in all subjects. Higher risk of HCC was associated with elevated levels of total and individual primary bile acid species and the ratios of taurine-over glycine-conjugated bile acids, as well as with reduced ratios of secondary over primary bile acid species (Supplementary Figure S2).

To assess the potential impact of HCC status and progression on the metabolism of bile acids, a sensitivity analysis was conducted after excluding all cases diagnosed within two years after blood draw and their matched controls. In this subgroup analysis including 78 case–control pairs, the bile acid–HCC risk associations were not materially changed. Higher risk of HCC was associated with elevated levels of total and individual primary bile acid species and the ratios of taurine-over glycine-conjugated bile acids, as well as with reduced ratios of secondary over primary bile acid species (Supplementary Figure S3).

## 4. Discussion

The present study demonstrated that individuals who developed HCC had significantly higher levels of total and individual major primary bile acids in sera collected approximately four years prior to the diagnosis of HCC than those who remained free of cancer in the Singapore Chinese Health Study. More interestingly, higher ratios of taurine-conjugated over glycine-conjugated major primary and secondary bile acids, specifically the TCDCA/GCDCA ratio and the TDCA/GDCA ratio, were associated with significantly higher risk of HCC, after adjustment for potential confounders, suggesting that the altered conjugation process may contribute to the development of HCC on top of their levels. The present study demonstrated that HCC cases had significantly lower ratios of major secondary bile acid species over their parent primary bile acid species (i.e., DCA/CA ratio, LCA/CDCA ratio, and UDCA/CDCA ratio) than controls, suggesting that the reduction in gut microbiota capable of producing secondary bile acids may play a significant role in the development of HCC. We also observed a statistically significant inverse association between the ratio of LCA over CDCA and HCC risk, suggesting that LCA may offer a protective effect opposite to CDCA on the development of HCC in humans. These results were consistent with findings from in vitro studies that showed LCA, without the presence of CDCA, inhibited cell growth of several cancer cell lines including breast cancer [40], neuroblastoma [41], and prostate cancer [42].

Serum concentrations of primary bile acids may reflect their synthesis and metabolism in the liver and the transportation from the liver to the gallbladder. Any factors that alter the homeostasis of bile acids may have an impact on overall liver health and chronic liver toxicity. Several studies that previously examined pre-diagnostic blood levels of bile acids in relation to HCC risk produced similar results to ours, but in different study populations with different underlying risk factors for HCC. In a recent publication by Petrick et al. [23], higher levels of major primary bile acids including GCA, TCA, GCDCA, and TCDCA in pre-diagnostic sera were associated with significantly increased risk of HCC in Chinese subjects in Taiwan who all were chronic carriers of HBV or HCV or both. In another recent study by Loftfield et al., using an untargeted metabolomic approach, elevated levels of serum GCA and GCDCA were found to be associated with significantly elevated risk of liver cancer incidence or fatal liver disease in male smokers of the Alpha-Tocopherol, Beta-Carotene Cancer Prevention (ATBC) cohort in Finland [24]. A retrospective analysis of more than 2,200 patients with chronic HBV infection found that patients with persistently elevated serum levels of total bile acids had significantly higher hazard ratios of developing HCC in China [26]. In a longitudinal study of 33 individuals with positive HBsAg with repeated serum samples collected at baseline and repeated at 6-month intervals for 24 months prior to HCC diagnosis (11 developed HCC and 22 were free of HCC as controls), the relative abundances of GCA, TCA, GCDCA, and TCDCA were significantly higher in persons who developed HCC than those of controls at different corresponding time points [27]. The results from previous studies in populations with chronic HBV or HCV infections or smokers were consistent with our findings [23,24]. Importantly, our study demonstrates that the positive association between serum bile acids and HCC risk was present in individuals without any chronic infection with HBV or HCV, which suggests MAFLD as a likely underlying risk factor for HCC. In addition, our analysis also revealed that elevated bile acids were associated with the risk of HCC with more than two years of follow-up. These results suggest that bile acids may play an important role in the progression of underlying liver diseases that lead to HCC.

The elevated serum bile acids may be the results of a compromising liver due to the underlying diseases. Previous studies have shown that serum bile acids were higher in patients chronically infected with HBV/HCV [21,43,44] and increased with advancing stage of liver disease in a dose-dependent manner [45,46,47]. To minimize the potential impact of underlying liver disease on the metabolism of bile acids, we conducted several sensitivity analyses on subsets of subjects by (1) excluding any individuals known to be infected with HBV and/or HCV, and (2) eliminating HCC cases (and their matched controls) whose assessment of bile acids was done less than 24 months prior to the diagnosis of HCC. The similarity of the results from these sensitivity analyses to those derived from the entire data set did not support the hypothesis that the observed positive associations between serum bile acids and HCC risk may be completely due to the underlying liver disease.

Under normal conditions, unconjugated bile acids activate the nuclear receptor farnesoid X receptor (FXR) in the liver to reduce hepatic synthesis of bile acids, which would maintain the homeostasis of bile acids [14,48]. In addition, FXR plays a critical role in regulating hepatic and gastrointestinal inflammation and immune response [14,48]. For example, FXR may antagonize nuclear factor kappa-B (NF-kB) signaling, resulting in a reduction of pro-inflammatory cytokine production in the liver, and may be expressed in macrophages to repress pro-inflammatory cytokine expression [48,49,50]. In contrast, conjugated bile acids have less potential to activate FXR than unconjugated bile acids [14]. High levels of conjugated primary bile acids may not be able to activate FXR as readily in patients with MAFLD or fibrosis. In combination with a potentially dysregulated microbiome, conjugated bile acids may lead to dysregulation of FXR, resulting in elevated production of bile acids in the liver, NF-kB pathway activation, hepatic inflammation, and carcinogenesis [14].

Besides FXR, bile acids are ligands for other transcription factors including G protein-coupled bile acid receptor (TGR5), vitamin D3 receptor (VDR), pregnane X receptor (PXR), and constitutive androstane receptor (CAR) [14]. For example, TGR5 is recognized as a potential target for the treatment of metabolic disorders such as type 2 diabetes. The activation of TGR5 can enhance energy expenditure and lower pro-inflammatory cytokine levels [51]. These experimental data show the complex role of bile acids in the signaling pathways that impact cell proliferation and apoptosis.

To our knowledge, this is the first study to show that the ratio of taurine-conjugated bile acids, especially TCA and TCDCA, over their glycine-conjugated bile acids, were significantly associated with increased risk of HCC, suggesting that the former may have a stronger effect on HCC than the latter. The differential effect of taurine- vs. glycine-conjugated bile acids on HCC risk in humans has not been examined in prior studies. Our study found that the highest tertile of the TCDCA/GCDCA ratio was associated with a statistically significant 4-fold increased risk of HCC than the lowest tertile. These findings of positive associations between the ratios of taurine-over glycine-conjugated major primary and secondary bile acids and HCC risk provided further support to the role of altered bile acid metabolism on the development of HCC in humans because these ratios are less likely to be impacted by the compromising liver function due to underlying liver disease as they are based on the same parent bile acids. Taurine-conjugated bile acids have been shown to promote liver cirrhosis via upregulating Toll-like receptor 4 expression, and to increase intestinal permeability and render dysfunction of the intestinal barrier [52]. Additionally, TCA, but not GCA, has been shown to cause overgrowth of the bacterium *Bilophila wadsworthia*, which can elicit inflammation, leading to higher glucose dysmetabolism and hepatic steatosis [53]. Experimental studies have also shown that a high-fat diet significantly increased taurine-conjugated bile acid concentration in serum by more than 100-fold compared to normal diet in mice [53], and milk fats significantly promoted taurine conjugation of bile acids along with a bloom of intestinal bacteria [54]. These data suggest that a high-fat diet may enhance the production and activity of hepatic enzymes for taurine–bile acid conjugation or increase the reabsorption and circulation of taurine-conjugated bile acids. Further studies are warranted to understand the effect and determinants of elevated taurine-conjugated bile acids on HCC and other liver diseases. The ratios of taurine over glycine bile acids may be developed and ultimately serve as biomarkers for monitoring the risk and disease progression of MAFLD.

Our study has several strengths. The present study was conducted in a population-based cohort representative of a general population, which may overcome the limited generalizability of findings in previous studies. We used the state-of-the-art technology that quantified a comprehensive panel of 35 unique bile acids, more than double the number in any previous studies, which allowed us to conduct detailed analysis for the associations of HCC risk with individual, summed, and ratios of bile acids. We employed a prospective study design in which the collection of serum samples for measurement of bile acids was done, on average, four years prior to diagnosis of HCC, minimizing the impact of the progression and presence of HCC on circulating levels of measured bile acids. The present study was the first to examine the differential effect of taurine-conjugated bile acids as compared with glycine-conjugated bile acids on the risk of HCC incidence. Lastly, we were able to show that bile acids were associated with HCC, excluding participants who were HBsAg or HCV positive, indicating that bile acid metabolism may be implicated in MAFLD-driven HCC.

Our study also has several limitations. The measurement of bile acids was done in non-fasting serum samples collected at a random, single point of time. The non-differential misclassification due to intra-individual variation in bile acid over time may result in underestimated true association between bile acids studied and HCC risk. The hours from the last meal to blood draw was also adjusted for in the statistical analysis. We did not have anti-HCV serologic status in all study subjects. Given its low prevalence (only one out of 60 HCC cases was detected positive for anti-HCV), its potential impact on the bile acid–HCC risk association would be minimal. Given the small sample size of our study, we were unable to examine the impact of genetic polymorphisms on circulating bile acids and the risk of HCC. Lastly, our study was conducted in a Han Chinese population, which may limit the generalizability of our results to other populations. However, the consistent association between various measurements of bile acids and HCC risk in our study population as compared with a prior study in a Finnish population [24] may support a broad generalizability of findings to different populations.

## 5. Conclusions

In conclusion, our study clearly demonstrates a strong association between elevated serum concentrations of major primary bile acids measured approximately four years prior to diagnosis and greater risk of developing HCC. In addition, this is the first study to find that the effect of taurine-conjugated bile acids on HCC risk is stronger than their glycine-conjugated counterparts. The present study also shows a significant association between reduced relative abundance of secondary bile acids over primary bile acids and higher risk of HCC, implying that gut microbiota may play a significant role in the risk of HCC via the altered metabolism of bile acids. The findings of the present study, if confirmed in additional studies with larger sample sizes in diverse populations, may have public health implications and clinical utility. Bile acid levels and profiles may be used to identify individuals at high risk for HCC development and/or the progression of liver disease toward HCC. The modulation of bile acid metabolism, especially the taurine-over glycine-conjugated bile acid ratios, through dietary modification and altered gut microbiota may be an effective strategy for primary prevention against the development of MAFLD-related HCC in humans.

## Figures and Tables

**Table 1:** Baseline characteristics of hepatocellular carcinoma (HCC) cases and matched controls, The Singapore Chinese Health Study.

Characteristics	HCC Cases	Controls	*p*
N	100	100	
Age (years), mean (SD)	66.4 (7.1)	66.3 (6.9)	0.936
Female sex, N (%)	25 (25%)	25 (25%)	1.000
BMI (kg/m^2^), mean (SD)	24.2 (3.8)	23.8 (3.5)	0.461
Hours between last meal and blood draw, mean (SD)	4.8 (4.6)	5.3 (5.4)	0.555
Alcoholic drinks/week, N (%)			
Zero	75 (75%)	79 (79%)	0.192
1– <7	13 (13%)	16 (16%)	
7+	12 (12%)	5 (5%)	
Smoking Status, N (%)			
Never	44 (44%)	49 (49%)	0.553
Former	33 (33%)	34 (34%)	
Current	23 (23%)	17 (17%)	
HBsAg Status, N (%)			
Negative	60 (60%)	92 (92%)	<0.001
Positive	40 (40%)	8 (8%)	
History of diabetes, N (%)			
Yes	30 (30%)	12 (12%)	0.002
No	70 (70%)	88 (88%)	
Anti-HCV Status, N (%) ^a^			
Negative	59 (98%)	58 (98%)	1.00
Positive	1 (2%)	1 (2%)	

Chi-square for categorical variables, *t*-test for means. ^a^ Only measured for 119 participants.

**Table 2:** Geometric means of major primary and secondary bile acids and molar ratios of the secondary over primary bile acids in hepatocellular carcinoma (HCC) cases and controls, The Singapore Chinese Health Study.

	Geometric Mean (95%CI) ^b^		
Major Bile Acid Species ^a^	HCC Cases	Controls	*p*
Number of subjects	100	100	
Primary Bile Acids			
CA species (nM)	1678 (1367, 2061)	648 (527, 796)	**<0.001**
CDCA species (nM)	9644 (8031, 11580)	3499 (2914, 4202)	**<0.001**
Summed major primary bile acids (nM) ^c^	11612 (9722, 13871)	4329 (3624, 5171)	**<0.001**
Secondary Bile Acids			
DCA species (nM)	2026 (1676, 2448)	1649 (1365, 1993)	0.134
LCA species (nM)	1014 (845, 1217)	828 (690, 993)	0.125
UDCA species (nM)	518 (429, 625)	375 (311, 453)	**0.018**
Summed major secondary bile acids (nM) ^d^	4589 (4029, 5226)	3356 (2946, 3822)	**0.001**
Molar ratio of secondary over primary bile acids			
DCA species/CA species ratio	1.21 (0.96, 1.51)	2.55 (2.03, 3.19)	**<0.001**
LCA species/CDCA species ratio	0.11 (0.08, 0.14)	0.24 (0.18, 0.31)	**<0.001**
UDCA species/CDCA species ratio	0.05 (0.04, 0.07)	0.11 (0.09, 0.13)	**<0.001**

^a^ See specific bile acids included in the major bile acid species in Supplementary Table S1. CA, cholic acid; CDCA, chenodeoxycholic acid; DCA, deoxycholic acid; LCA, lithocholic acid; and UDCA, ursodeoxycholic acid. ^b^ Derived from analysis of variance (ANOVA). *p* < 0.05 is in bold. ^c^ Sum of CA species and CDCA species. ^d^ Sum of DCA species, LCA species, and UDCA species.

**Table 3:** Major primary and secondary individual bile acids and the molar ratios of secondary over primary bile acids in relation to risk of developing hepatocellular carcinoma, The Singapore Chinese Health Study.

Major Bile Acid Species ^a^	Odds Ratio (95% CI) ^b^ by Bile Acid in Tertile			
	1st	2nd	3rd	*P*_trend_
Primary Bile Acids				
CA species	1	1.14 (0.34, 3.81)	**6.09 (1.75, 21.21)**	**0.001**
CDCA species	1	3.41 (0.76, 15.28)	**30.11 (5.88, 154.31)**	**<0.001**
Summed major primary bile acids ^c^	1	**5.68 (1.35, 23.92)**	**32.59 (6.04, 175.84)**	**<0.001**
Secondary Bile Acids				
DCA species	1	0.49 (0.18, 1.35)	2.02 (0.83, 4.93)	0.105
LCA species	1	0.64 (0.24, 1.74)	1.22 (0.52, 2.83)	0.575
UDCA species	1	1.17 (0.39, 3.5)	**3.63 (1.26, 10.43)**	**0.013**
Summed major secondary bile acids ^d^	1	1.67 (0.66, 4.24)	2.5 (0.98, 6.39)	0.055
Molar ratio of secondary over primary bile acids				
DCA species/CA species ratio	1	0.58 (0.26, 1.30)	0.37 (0.14, 1.00)	**0.042**
LCA species/CDCA species ratio	1	0.74 (0.31, 1.77)	**0.27 (0.09, 0.81)**	**0.027**
UDCA species/CDCA species ratio	1	**0.41 (0.17, 0.99)**	**0.29 (0.10, 0.82)**	**0.013**

^a^ See specific bile acids included in the major bile acid species in Supplementary Table S1. CA, cholic acid; CDCA, chenodeoxycholic acid; DCA, deoxycholic acid; LCA, lithocholic acid; and UDCA, ursodeoxycholic acid. ^b^ Derived from conditional logistic regression models including following covariates: HBsAg status, alcohol intake, cigarette smoking status, history of diabetes, body mass index, and time interval from the last meal to blood draw. Odds ratios with 95% confidence intervals (CIs) excluding one and *p* < 0.05 are in bold. ^c^ Sum of CA species and CDCA species. ^d^ Sum of DCA species, LCA species, and UDCA species.

**Table 4:** Geometric means of free and conjugated bile acids and the molar ratios of taurine-over glycine-conjugated bile acids in hepatocellular carcinoma (HCC) cases and controls, The Singapore Chinese Health Study.

	Geometric Mean (95% CI) ^b^		
Bile acid ^a^	HCC Cases	Controls	*p*
Number of subjects	100	100	
Primary Bile Acids			
CA (nM)	100 (72, 140)	95 (68, 132)	0.807
GCA (nM)	796 (580, 1094)	211 (153, 289)	**<0.001**
TCA (nM)	169 (118, 243)	36 (25, 52)	**<0.001**
TCA/GCA ratio	0.21 (0.15, 0.30)	0.17 (0.12, 0.24)	0.375
CDCA (nM)	920 (751, 1127)	825 (673, 1010)	0.454
GCDCA (nM)	6234 (5018, 7743)	1883 (1516, 2339)	**<0.001**
TCDCA (nM)	675 (489, 932)	110 (80, 152)	**<0.001**
TCDCA/GCDCA ratio	0.11 (0.09, 0.14)	0.06 (0.05, 0.07)	**<0.001**
Secondary Bile Acids			
DCA (nM)	629 (518, 763)	773 (637, 937)	0.14
GDCA (nM)	839 (638, 1105)	566 (430, 745)	**0.048**
TDCA (nM)	124 (89, 171)	45 (32, 62)	**<0.001**
TDCA/GDCA ratio	0.15 (0.12, 0.19)	0.08 (0.06, 0.1)	**<0.001**
UDCA (nM)	243 (184, 319)	145 (110, 190)	**0.010**
GUDCA (nM)	15 (12.3, 18.2)	11.8 (9.7, 14.3)	0.088
TUDCA (nM)	23.2 (18.8, 28.6)	14.2 (11.5, 17.6)	**0.002**
TUDCA/GUDCA ratio	1.55 (1.23, 1.94)	1.21 (0.97, 1.52)	0.135

^a^ CA, cholic acid; GCA, glyco-cholic acid; TCA, tauro-cholic acid; CDCA, chenodeoxycholic acid; GCDCA, glyco-chenodeoxycholic acid; TCDCA, tauro-chenodeoxycholic acid; DCA, deoxycholic acid; GDCA, glyco-deoxycholic acid; TDCA, tauro-deoxycholic acid; UDCA, ursodeoxycholic acid; GUDCA, glyco-ursodeoxycholic acid; and TUDCA, tauro-ursodeoxycholic acid. ^b^ Derived from analysis of variance (ANOVA). *p* < 0.05 is in bold; CI, confidence interval.

**Table 5:** Free and conjugated specific bile acids and the molar ratios of taurine-over glycine-conjugated bile acids in relation to risk of developing hepatocellular carcinoma, The Singapore Chinese Health Study.

	Odds Ratio (95% CI) ^b^ by Bile Acid in Tertile			
Bile Acid ^a^	1st	2nd	3rd	*P*_trend_
Primary Bile Acids				
CA	1	0.61 (0.18, 2.08)	1.14 (0.38, 3.42)	0.481
GCA	1	1.31 (0.35, 4.97)	**6.76 (2.04, 22.41)**	**<0.001**
TCA	1	1.93 (0.48, 7.82)	**14.94 (3.43, 65.05)**	**<0.001**
TCA/GCA ratio	1	3.09 (1.12, 8.53)	2.97 (0.91, 9.72)	0.091
CDCA	1	1.40 (0.56, 3.51)	1.41 (0.57, 3.46)	0.476
GCDCA	1	5.56 (0.95, 32.65)	**57.22 (7.47, 438.35)**	**<0.001**
TCDCA	1	3.75 (0.66, 21.3)	**16.69 (3.11, 89.48)**	**<0.001**
TCDCA/GCDCA ratio	1	1.60 (0.47, 5.50)	**4.34 (1.38, 13.71)**	**0.006**
Secondary Bile Acids				
DCA	1	1.05 (0.46, 2.37)	0.64 (0.27, 1.52)	0.341
GDCA	1	0.94 (0.36, 2.48)	**3.86 (1.46, 10.23)**	**0.009**
TDCA	1	0.4 (0.1, 1.52)	2.52 (0.91, 6.98)	**0.008**
TDCA/GDCA ratio	1	2.43 (0.78, 7.60)	**3.01 (1.10, 8.20)**	**0.039**
UDCA	1	1.47 (0.5, 4.27)	**3.81 (1.46, 9.95)**	**0.006**
GUDCA	1	0.76 (0.29, 1.97)	2.36 (0.92, 6.05)	0.084
TUDCA	1	1.41 (0.52, 3.83)	1.74 (0.68, 4.46)	0.256
TUDCA/GUDCA ratio	1	0.95 (0.39, 2.31)	0.82 (0.29, 2.30)	0.709

^a^ GCA, glycol-cholic acid; TCA, tauro-cholic acid; GCDCA, glyco-chenodeoxycholic acid; TCDCA, tauro-chenodeoxycholic acid; GDCA, glyco-deoxycholic acid; TDCA, tauro-deoxycholic acid; GUDCA, glyco-ursodeoxycholic acid; and TUDCA, tauro-ursodeoxycholic acid. ^b^ Derived from conditional logistic regression models including the following covariates: HBsAg status, alcohol intake, cigarette smoking status, history of diabetes, body mass index, and time interval from the last meal to blood draw. Odds ratios with 95% confidence intervals (CIs) excluding one and *p* < 0.05 are in bold.

## Data Availability

The data that support the findings of this study are available from the corresponding author upon reasonable request.

## Supplementary Materials

The following are available online at https://www.mdpi.com/article/10.3390/cancers13112648/s1, Figure S1: Spearman correlation coefficients between bile acids among all control subjects, The Singapore Chinese Health Study, Figure S2: Odds ratio (95% confidence interval) of hepatocellular carcinoma associated with doubling concentrations or ratios of bile acids among subjects who did not test positive for both hepatitis B surface antigen and antibodies to hepatitis C virus (53 case-control pairs), The Singapore Chinese Health Study, Figure S3: Odds ratio (95% confidence interval) of hepatocellular carcinoma associated with doubling concentrations or ratios of bile acids in subjects with at least 2 years of follow-up after blood draw (78 case-control pairs), The Singapore Chinese Health Study, Table S1: Classification of measured bile acids, Table S2: Geometric means of other individual bile acids in hepatocellular carcinoma (HCC) cases and controls, The Singapore Chinese Health Study, Table S3: Odds ratios (95% CI) of hepatocellular carcinoma development by tertile or doubling concentrations of other individual bile acids, The Singapore Chinese Health Study, Table S4: Geometric means (nM) of bile acid species by different categories of selected baseline characteristics among control subjects, The Singapore Chinese Health Study.
