# Supplementary material for: Association between Pre-Diagnostic Serum Bile Acids and Hepatocellular Carcinoma: The Singapore Chinese Health Study
Source: Cancers (Basel). 2021 May 28;13(11):2648. doi: 10.3390/cancers13112648 (PMC8198655; doi:10.3390/cancers13112648)
Supplement: Supplementary file 1 [file cancers-13-02648-s001.zip › cancers-1230252-supplementary.pdf]

Supplementary Tables and Figures: Association between pre-diagnostic serum bile acids and hepatocellular carcinoma: The Singapore Chinese Health Study

Claire E. Thomas[1,2], Hung N. Luu[1,2], Renwei Wang[2], Guoxiang Xie[3], Jennifer Adams-Haduch[2], Aizhen Jin[4], Woon-Puay Koh[4], Wei Jia[3], Jaideep Behari[2,5], Jian-Min Yuan[1,2*]

Authors' Affiliations:

[1] Department of Epidemiology, Graduate School of Public Health, University of Pittsburgh, Pittsburgh, PA, USA
[2] UPMC Hillman Cancer Center, University of Pittsburgh, Pittsburgh, PA, USA
[3] University of Hawaii Cancer Center, Honolulu, Hawaii, USA
[4] Healthy Longevity Translational Research Programme, Yong Loo Lin School of Medicine, National University of Singapore.
[5] Department of Medicine, Division of Gastroenterology, Hepatology, and Nutrition, School of Medicine, University of Pittsburgh, Pittsburgh, PA, USA

Corresponding Author: Jian-Min Yuan (yuanj@upmc.edu). University of Pittsburgh Medical Center (Shadyside) Cancer Pavilion, 5150 Centre Avenue - Suite 4C, Room 470, Pittsburgh, PA 15232, Primary Phone: 412-864-7889 Fax: 412-864-7838.

Supplementary Table S1: Classification of measured bile acids

Bile acid species	Bile Acid (abbreviation)	Primary or secondary bile acid	Grouping of Conjugation (taurine, glycine, or other)
CA	Cholic acid (CA)	Primary	Unconjugated
CA	Glycocholic acid (GCA)	Primary	Conjugated (glycine)
CA	Norcholic acid (NorCA)	Primary	Conjugated (other)
CA	Taurocholic acid (TCA)	Primary	Conjugated (taurine)
CA	β-cholic acid (βCA)	Primary	Conjugated (other)
CA	β-ursocholic acid (βUCA)	Primary	Conjugated (other)
CDCA	Chenodeoxycholic acid (CDCA)	Primary	Unconjugated
CDCA	Chenodeoxycholic acid 24-glucuronide (CDCA-24G)	Primary	Conjugated (other)
CDCA	Glycochenodeoxycholic acid (GCDCA)	Primary	Conjugated (glycine)
CDCA	Taurochenodeoxycholic acid (TCDCA)	Primary	Conjugated (taurine)
CDCA	7-ketolithocholic acid (7-ketoLCA)	Primary	Conjugated (other)
DCA	Deoxy-cholic acid (DCA)	Secondary	Unconjugated
DCA	Glycodeoxycholic acid (GDCA)	Secondary	Conjugated (glycine)
DCA	Isodeoxycholic acid (isoDCA)	Secondary	Conjugated (other)
DCA	Nordeoxycholic acid (NorDCA)	Secondary	Conjugated (other)
DCA	Taurodeoxycholic acid (TDCA)	Secondary	Conjugated (taurine)
DCA	12-ketolithocholic acid (12-ketoLCA)	Secondary	Conjugated (other)
LCA	6,7-dikotolithocholic acid (6,7-dikotoLCA)	Secondary	Conjugated (other)
LCA	Allolithocholic acid (alloLCA)	Secondary	Conjugated (other)
LCA	Glycolithocholic acid (GLCA)	Secondary	Conjugated (glycine)
LCA	Glycolithocholic acid-3-sulfate (GLCA-3S)	Secondary	Conjugated (glycine)
LCA	Isolithocholic acid (isoLCA)	Secondary	Conjugated (other)
LCA	Lithocholic acid (LCA)	Secondary	Unconjugated
LCA	Lithocholic acid-3-sulfate (LCA-3S)	Secondary	Conjugated (other)
UDCA	Glycoursodeoxycholic acid (GUDCA)	Secondary	Conjugated (glycine)
UDCA	Tauroursodeoxycholic acid (TUDCA)	Secondary	Conjugated (taurine)
UDCA	Ursodeoxycholic acid (UDCA)	Secondary	Unconjugated
UDCA	β-ursodeoxycholic acid (βUDCA)	Secondary	Conjugated (other)
Other minor species	Glycohydrocholic acid (GHCA)	Primary	Conjugated (glycine)
Other minor species	Hydrocholic acid (HCA)	Primary	Unconjugated
Other minor species	Taurohydrocholic acid (THCA)	Primary	Conjugated (taurine)
Other minor species	Glycol-hyodeoxycholic acid (GHDCA)	Secondary	Conjugated (glycine)
Other minor species	Hyodeoxycholic acid (HDCA)	Secondary	Unconjugated
Other minor species	Tauro-hyodeoxycholic acid (THDCA)	Secondary	Conjugated (taurine)
Other minor species	β-hyodeoxycholic acid (βHDCA)	Secondary	Conjugated (other)

Supplementary Table S2. Geometric means of other individual bile acids in hepatocellular carcinoma (HCC) cases and controls, The Singapore Chinese Health Study

	Geometric mean (95% CI)[a]		
**Bile acid**	**HCC Cases**	**Controls**	***P***
Minor CA species			
NorCA	18.78 (15.55, 22.68)	20.54 (17.01, 24.8)	0.512
βCA	10.73 (8.52, 13.52)	9.92 (7.87, 12.49)	0.635
βUCA	8.41 (6.79, 10.41)	7.69 (6.21, 9.51)	0.559
Minor CDCA species			
CDCA-24G	**411.2 (295.65, 571.93)**	**106.65 (76.68, 148.34)**	**<0.001**
7-ketoLCA	4.69 (3.84, 5.73)	4.73 (3.87, 5.77)	0.96
Minor DCA species			
isoDCA	16.33 (13.3, 20.05)	19.66 (16.01, 24.14)	0.212
NorDCA	2.68 (2.2, 3.28)	2.02 (1.65, 2.46)	0.05
12-ketoLCA	5.21 (4.19, 6.49)	4.28 (3.44, 5.33)	0.214
Minor LCA species			
6,7-dikotoLCA	35.16 (27.98, 44.17)	35.15 (27.97, 44.16)	0.999
isoLCA	12.49 (10.16, 15.36)	11.83 (9.62, 14.55)	0.716
alloLCA	18.61 (15.28, 22.65)	18.33 (15.05, 22.31)	0.915
LCA-3S	9.33 (8.59, 10.14)	8.98 (8.26, 9.76)	0.52
GLCA-3S	720.79 (560.59, 926.78)	546.06 (424.69, 702.12)	0.127
Minor UDCA species			
βUDCA	93.11 (68.58, 126.41)	99.9 (73.58, 135.64)	0.75
Other minor species			
HCA	4.58 (3.62, 5.8)	3.81 (3.01, 4.83)	0.281
GHCA	5.27 (4.3, 6.45)	4.08 (3.34, 5)	0.083
THCA	8.74 (6.44, 11.86)	7.16 (5.27, 9.71)	0.366
HDCA	61.75 (44.93, 84.87)	78.9 (57.41, 108.43)	0.287
GHDCA	3.94 (3.3, 4.71)	3.55 (2.98, 4.24)	0.422
THDCA	8.37 (6.32, 11.08)	7.87 (5.94, 10.42)	0.76
βHDCA	8.53 (6.31, 11.53)	8.71 (6.45, 11.77)	0.923

[a] Derived from analysis of variance (ANOVA). $P < 0.05$ is in bold. CI, confidence interval.

Supplementary Table S3. Odds ratios (95% CI) of hepatocellular carcinoma development by tertile or doubling concentrations of other individual bile acids, The Singapore Chinese Health Study

	Odds ratio (95% CI)[a], SCHS				
**Bile Acid**	**Tertile 1**	**Tertile 2**	**Tertile 3**	**p-trend**	**Doubling concentration[b]**
Minor CA species					
NorCA	1	0.57 (0.22, 1.47)	0.88 (0.33, 2.33)	0.642	0.94 (0.71, 1.24)
βCA	1	1.6 (0.64, 4.03)	1.45 (0.54, 3.86)	0.476	1.06 (0.83, 1.36)
βUCA	1	2.24 (0.87, 5.82)	2.54 (0.9, 7.16)	0.063	1.09 (0.85, 1.41)
Minor CDCA species					
CDCA-24G	1	2.24 (0.63, 7.91)	**10.97 (2.88, 41.78)**	**<0.001**	**1.55 (1.23, 1.95)**
7-ketoLCA	1	1.16 (0.51, 2.66)	1.8 (0.76, 4.28)	0.19	1.24 (0.95, 1.62)
Minor DCA species					
isoDCA	1	0.85 (0.38, 1.89)	0.93 (0.36, 2.4)	0.858	0.93 (0.66, 1.3)
NorDCA	1	0.59 (0.22, 1.59)	2.04 (0.65, 6.43)	0.219	1.27 (0.94, 1.72)
12-ketoLCA	1	0.94 (0.34, 2.59)	1.44 (0.51, 4.08)	0.463	1.13 (0.88, 1.44)
Minor LCA species					
6,7-dikotoLCA	1	**2.88 (1.06, 7.85)**	1.32 (0.51, 3.4)	0.516	1.07 (0.85, 1.36)
isoLCA	1	1.42 (0.57, 3.54)	1.05 (0.45, 2.48)	0.974	1.1 (0.86, 1.39)
alloLCA	1	0.8 (0.32, 2.02)	1.21 (0.44, 3.32)	0.672	1.07 (0.81, 1.41)
LCA-3S	1	0.64 (0.24, 1.71)	0.67 (0.22, 2.09)	0.484	1.24 (0.61, 2.51)
GLCA-3S	1	0.79 (0.3, 2.04)	1.42 (0.61, 3.28)	0.396	1.13 (0.92, 1.39)
Minor UDCA species					
βUDCA	1	2.04 (0.82, 5.07)	2.43 (0.86, 6.88)	0.086	1.02 (0.86, 1.21)
Other minor species					
HCA	1	1.83 (0.67, 4.99)	2.25 (0.77, 6.58)	0.153	1.24 (0.93, 1.64)
GHCA	1	2.1 (0.75, 5.91)	2.33 (0.79, 6.93)	0.152	**1.45 (1.05, 2.01)**
THCA	1	1.17 (0.38, 3.6)	2.07 (0.6, 7.15)	0.242	1.1 (0.86, 1.42)
HDCA	1	0.5 (0.18, 1.39)	0.61 (0.2, 1.93)	0.354	0.86 (0.69, 1.07)
GHDCA	1	**3.11 (1.07, 9.01)**	**3.25 (1.13, 9.38)**	**0.044**	**1.39 (1, 1.92)**
THDCA	1	0.93 (0.31, 2.79)	0.82 (0.15, 4.64)	0.827	1.09 (0.8, 1.49)
βHDCA	1	1.43 (0.53, 3.86)	0.89 (0.29, 2.72)	0.794	0.96 (0.79, 1.17)

[a]Conditional logistic regression adjusted for HBsAg, drinking status, smoking status, diabetes, BMI, and time between last meal and blood draw;

[b]Log_2 Odds Ratio and 95% confidence interval (CI).

Supplementary Table S4. Geometric means (nM) of bile acid species by different categories of selected baseline characteristics among control subjects, The Singapore Chinese Health Study

	N	CA Species	CDCA species	DCA species	LCA species	UDCA species	Summed major primary bile acids	Summed major secondary bile acids
Total	100	648	3499	1649	828	375	4329	3356
Age								
<63	29	595	2874	1908	818	441	3638	3661
63-<70	37	602	3537	1466	808	377	4330	3190
70+	34	755	4090	1654	859	326	5019	3292
P for trend		0.289	0.123	0.545	0.806	0.152	0.131	0.510
Sex								
Male	75	663	3675	1618	784	399	4530	3330
Female	25	605	3020	1743	975	311	3776	3435
P for difference		0.667	0.347	0.710	0.245	0.200	0.350	0.824
BMI								
<23	46	**522**	**3030**	**1360**	886	423	**3703**	3219
23-<25	25	**604**	**3270**	**1657**	678	310	**4035**	2943
25+	29	**970**	**4661**	**2229**	882	366	**5891**	4014
P for trend		**0.005**	**0.050**	**0.015**	0.852	0.387	**0.023**	0.166
Alcohol Intake (drinks per day)								
0	79	667	3484	1774	845	345	4324	3394
<1	16	593	3716	1174	813	563	4462	3322
1+	5	538	3086	1542	630	380	3998	2903
P for trend		0.508	0.953	0.188	0.492	0.145	0.946	0.624
Smoking Status								
Never	49	633	3388	1636	743	410	4191	3293
Former	34	796	3874	1859	961	369	4887	3789
Current	17	458	3133	1326	840	300	3730	2779
P for trend		0.120	0.689	0.416	0.367	0.414	0.882	0.615
HBsAg								
Negative	92	647	3592	1722	810	387	4414	3399
Positive	8	660	2586	1004	1057	266	3457	2895
P for difference		0.952	0.324	0.087	0.376	0.229	0.432	0.474
Time b/w recent meal & blood draw								
<3 hours	55	747	**4259**	1668	786	403	**5186**	3396
3-<6 hours	19	569	**3321**	1621	885	357	**4032**	3264
6+ hours	26	527	**2399**	1631	880	334	**3111**	3338
P for trend		0.088	**0.006**	0.902	0.521	0.331	**0.009**	0.878
Diabetes status								
No	88	639	3450	1641	804	**352**	4284	3281
Yes	12	717	3879	1709	1027	**605**	4676	3960
P for difference		0.681	0.674	0.877	0.328	**0.035**	0.736	0.314

Supplementary Figure S1. Spearman correlation coefficients between bile acids among all control subjects, The Singapore Chinese Health Study

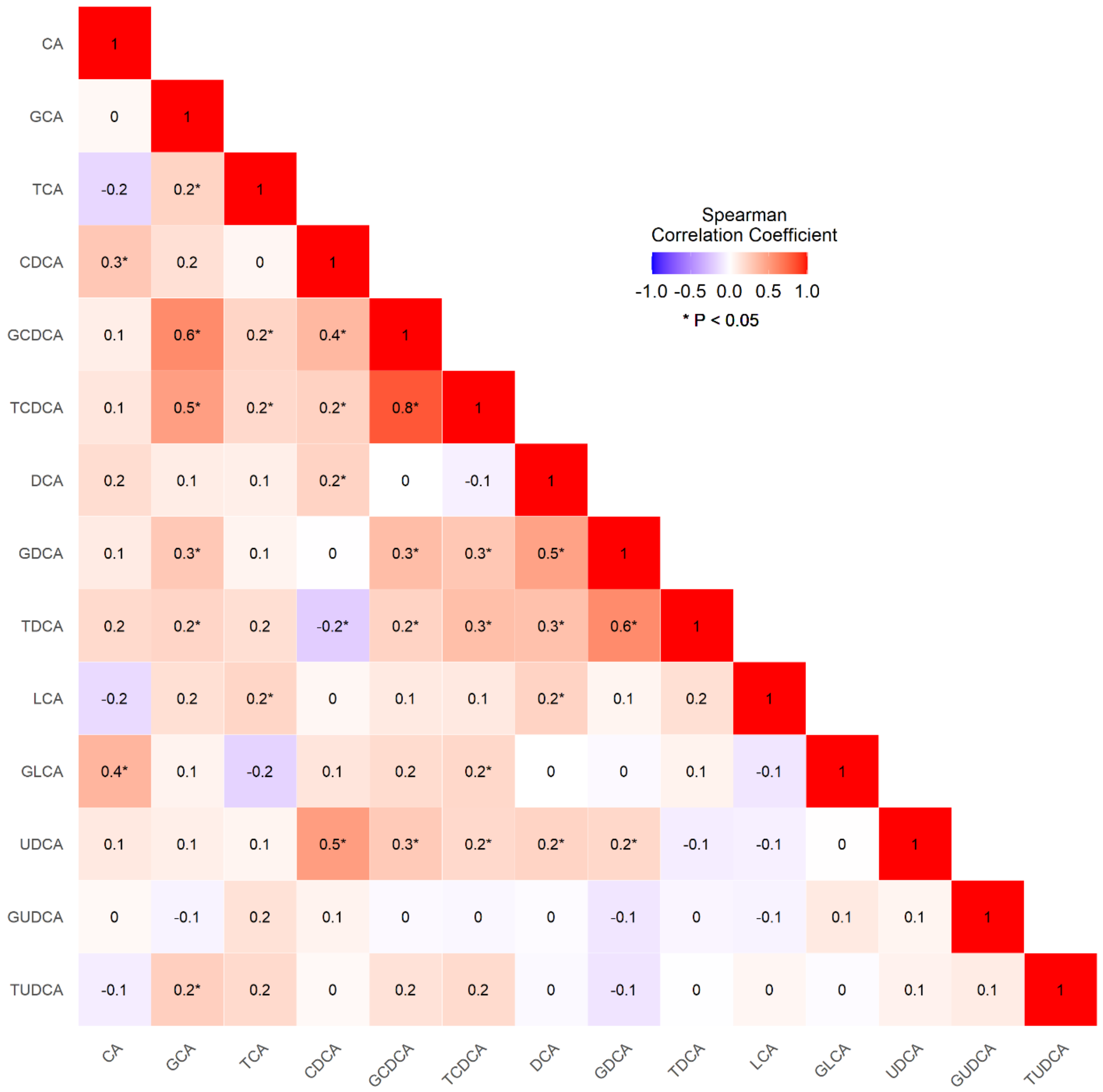

Supplementary Figure S2. Odds ratio (95% confidence interval) of hepatocellular carcinoma associated with doubling concentrations or ratios of bile acids among subjects who did not test positive for both hepatitis B surface antigen and antibodies to hepatitis C virus (53 case-control pairs), The Singapore Chinese Health Study

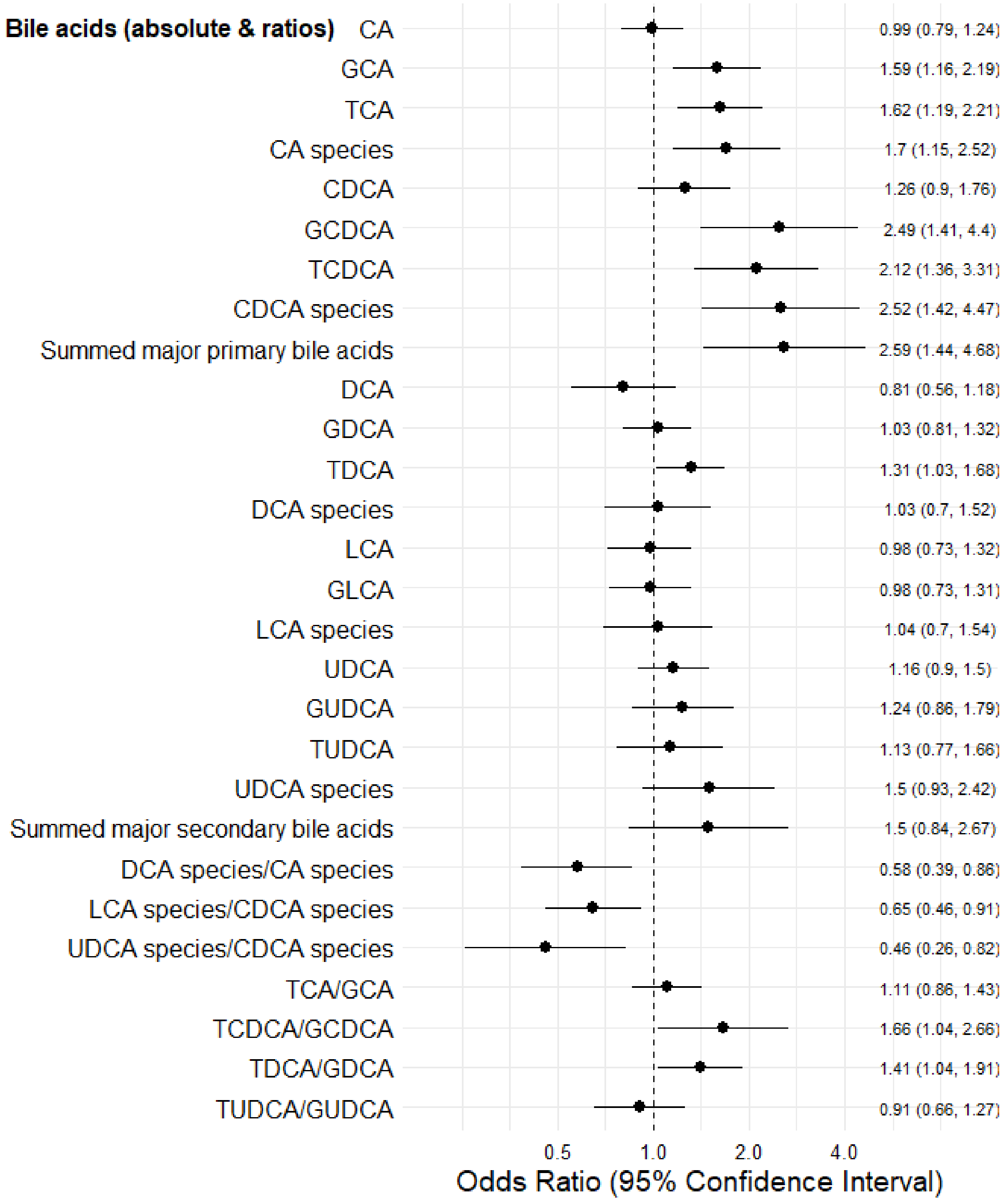

Conditional logistic regression adjusted for drinking status, smoking status, diabetes, BMI, and time between last meal and blood draw.

Supplementary Figure S3. Odds ratio (95% confidence interval) of hepatocellular carcinoma associated with doubling concentrations or ratios of bile acids in subjects with at least 2 years of follow-up after blood draw (78 case-control pairs), The Singapore Chinese Health Study

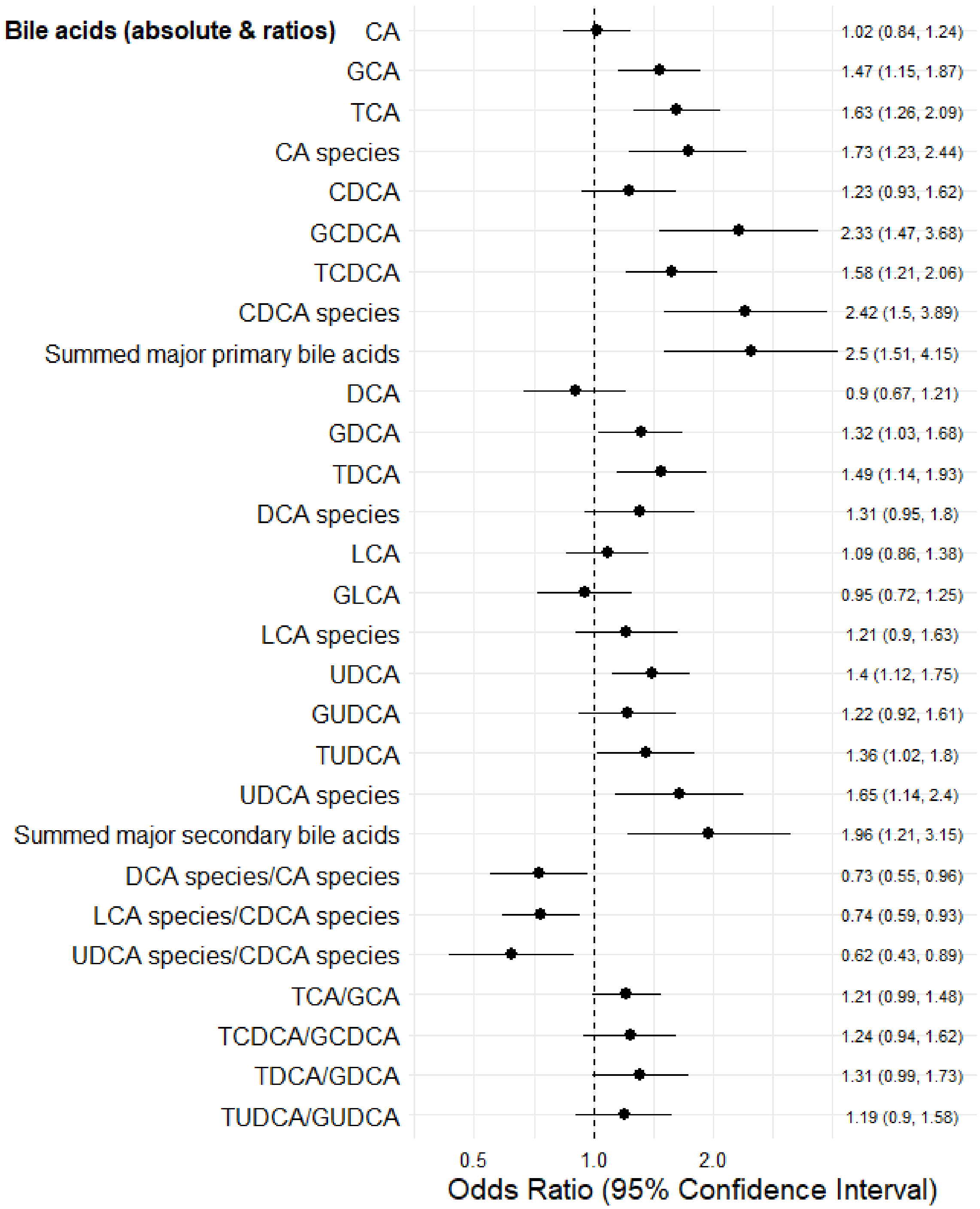

Conditional logistic regression adjusted for drinking status, smoking status, diabetes, BMI, HBsAg, and time between last meal and blood draw.
